# Behaviour of the Antitumor Agent Vanadocene Dichloride in Physiological and Therapeutic Media, Blood Plasma and Human Blood - An EPR Study

**DOI:** 10.1155/MBD.1997.207

**Published:** 1997

**Authors:** Jaromír Vinklárek, Ivan Pavlík, Zdenĕk Černošek

**Affiliations:** Department of General and Inorganic Chemistry Faculty of Chemical Technology University of Pardubice Pardubice CZ-532 10 Czech Republic

## Abstract

By means of EPR spectroscopy the behaviour of the vanadocene dichloride (**I**), Cp_2_VCl_2_ (Cp=η^5^-C_5_H_5_), in various deoxygenated and non-deoxygenated physiological media and therapeutic solution as well as in blood plasma and stabilized human blood was studied. On the basis of measured values of isotropic spectroscopic splitting factor g_iso_ and isotropic hyperfine coupling constant A_iso_, the vanadocene species, [Cp_2_V(H_2_O)Cl]^+^ (**II**; g_iso_= 1.985, |A_iso_| = 7.68 mT), [Cp_2_V(H_2_O)_2_]^2+^ (**III**; g_iso_= 1.983, |A_iso_| = 7.92 mT), [Cp_2_V(OH_2_ (**IV**; g_iso_= 1.991, |A_iso_| = 6.285 mT), [Cp_2_VCl(DMSO)]^+^ (**V**; g_iso_= 1.985, |A_iso_| = 7.69 mT), the vanadyl species [VO(DMSO)_5_]^2+^ (**VI**; g_iso_= 1.964, |A_iso_| = 10.78mT) and [VO(H_2_O)_5_]^2+^ (**VII**; g_iso_= 1.955, |A_iso_| = 11.56 mT) have been identified. From the measurements it follows that **I** does not react in its first coordination sphere with any component of a system used other than water and DMSO, resp. As to water-containing media, its behaviour is fully consistent with that of **I** in pure aqueous media. It was found the only vanadocene species present after application of the therapeutic solution of **I** into human blood to be **IV** not interacting in its first coordination sphere with any blood component.


### BEHAVIOUR OF THE ANTITUMOR AGENT VANADOCENE DICHLORIDE IN PHYSIOLOGICAL AND THERAPEUTIC MEDIA, BLOOD PLASMA AND HUMAN BLOOD - AN EPR STUDY

Jaromír Vinklárek, Ivan Pavlík,* and Zdeněk Černošek

Department of General and Inorganic Chemistry, Faculty of Chemical Technology, University of Pardubice, CZ-532 10 Pardubice, Czech Republic

### ABSTRACT

By means of EPR spectroscopy the behaviour of the vanadocene dichloride (I), $Cp_2VCl_2$ ($Cp = \eta^5$-$C_5H_5$), in various deoxygenated and non-deoxygenated physiological media and therapeutic solution as well as in blood plasma and stabilized human blood was studied. On the basis of measured values of isotropic spectroscopic splitting factor $g_{iso}$ and isotropic hyperfine coupling constant $A_{iso}$, the vanadocene species, $[Cp_2V(H_2O)Cl]^+$ (**II**; $g_{iso}$= 1.985, $|A_{iso}|$ = 7.68 mT), $[Cp_2V(H_2O)_2]^{2+}$ (**III**; $g_{iso}$= 1.983, $|A_{iso}|$ = 7.92 mT), $Cp_2V(OH)_2$ (**IV**; $g_{iso}$= 1.991, $|A_{iso}|$ = 6.285 mT), $[Cp_2VCl(DMSO)]^+$ (**V**; $g_{iso}$= 1.985, $|A_{iso}|$ = 7.69 mT), the vanadyl species $[VO(DMSO)_5]^{2+}$ (**VI**; $g_{iso}$= 1.964, $|A_{iso}|$ = 10.78mT) and $[VO(H_2O)_5]^{2+}$ (**VII**; $g_{iso}$= 1.955, $|A_{iso}|$ = 11.56 mT) have been identified. From the measurements it follows that **I** does not react in its first coordination sphere with any component of a system used other than water and DMSO, resp. As to water-containing media, its behaviour is fully consistent with that of **I** in pure aqueous media. It was found the only vanadocene species present after application of the therapeutic solution of I into human blood to be **IV** not interacting in its first coordination sphere with any blood component.

### INTRODUCTION

Vanadocene dichloride (I), $Cp_2VCl_2$ ($Cp = \eta^5$-$C_5H_5$), belongs to the class of metallocene dihalides, $Cp_2MX_2$ (M = Ti, V, Nb, Mo; X = halide), representing a novel family of potent antitumor organometallic agents [1,2]. Its antitumor activity has been discovered by Köpf-Maier and Köpf [3]. Vanadocene dichloride was shown to be characterized by marked antiproliferative effects against some cultured animal and human tumor cells (murine Ehrlich ascites tumor [4], human KB as well as HeLa tumor [1], HEp-2 human epidermoid carcinoma [5-7] ) or normal, not transformed cells (human embryonic fibroblasts [8] ) *in vitro* as well as by systemic antineoplastic activity against several experimental tumor systems (fluid and solid Ehrlich ascites tumor [3,8] or TA3Ha murine mammary adenocarcinoma [5-7] ) *in vivo*. Various biological experiments (such as electron energy loss-spectroscopy microanalysis showing the main accumulation of vanadium in nuclear heterochromatin [9,10], precursor incorporation studies revealing inhibition of nucleic acid synthesis activities [11], cytokinetic investigations revealing mitotic depressions in tumor cells treated with **I** *in vitro* [12] and *in vivo* [13], cytological and morphological analysis of histological and ultrastructural alterations of animal Ehrlich ascites tumor cells treated with **I** *in vivo* [14] and human embryonic fibroblasts treated with I *in vitro* [8] ) pointed to the nucleic acids, especially DNA, as probable primary intracellular target for **I**.

There is number of problems arising from the use of metallocene dichlorides as cytostatics. These are related to the knowledge of the nature of a genuine active metallocene species present in physiological and therapeutic media, to the understanding of the molecular mode of antitumor action of antitumor inhibiting metallocene dihalides. Thus, the tasks to be solved may be formulated as follows: *(i)* the elucidation of the nature of species arising from $Cp_2MX_2$ in aqueous, therapeutic and physiological media, *(ii)* the study of the interaction metallocene species - biomolecule (a) in the course of the transport into the tumor cell, (b) inside the tumor cell (molecular mechanism of the oncostatic action).

The metallocene complex **I** appears to be especially attractive for solving problems above. The paramagnetism of the central metal atom $d^1$ V(IV) and its nuclear spin ($^{51}$V: I=7/2, abundance 99.8%) make **I** to be a suitable EPR structural probe.

The following problems are formulated for this study: *(i)* to elucidate the nature of species arising from **I** in physiological and therapeutic media, *(ii)* to investigate the interaction of **I** with blood plasma and stabilized human blood. Therefore, the detailed knowledge of the aqueous chemistry of **I** seems to be a prerequisite to get an insight into the nature of interactions of **I** with components of physiological and therapeutic solutions as well as of interactions **I**-biomolecule both in extracellular medium and in tumor cells.

Recently, Toney and Marks [15] have described the hydrolysis kinetics and equilibria for **I** and proposed a scheme for its hydrolysis consisting of a sequence of aquation and dissociation equilibria (Scheme I). It was found that the V-Cp bonds exhibited the long-lasting hydrolytic stability at physiological pH thus preserving the structural integrity of the vanadocene unit $[Cp_2V]^{2+}$.

On the basis of combined results of the chloride potentiometry and pH titrimetry, Toney and Marks [15] anticipated not only quantitative loss of both chloride ligands of the molecule **I** in the blood plasma, but also the existence of aqua and hydroxo vanadocene complexes as prevailing forms of hydrolysis products. They also supposed the vanadocene dihydroxide **IV** to be a prevailing vanadocene species in the blood plasma.

SCHEME I - Hydrolysis of $Cp_2VCl_2$ [15]

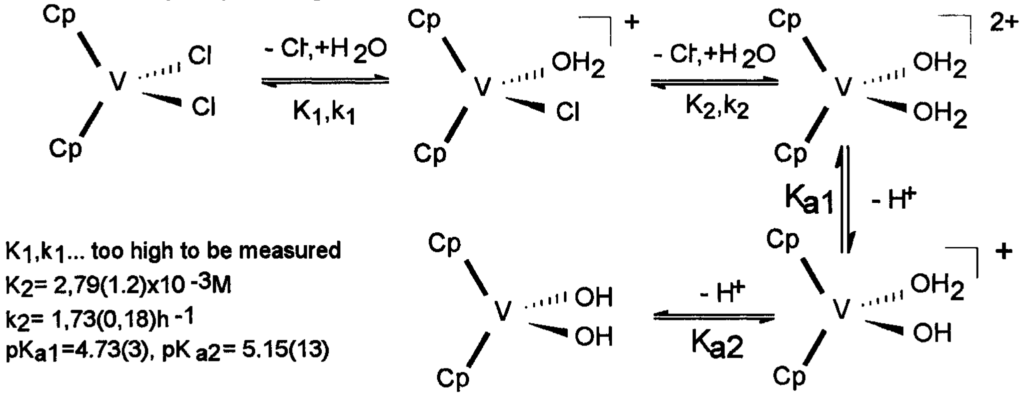

In a previous study [16] various hydrolysis species formed from **I** in strongly acidic, neutral and alkaline aqueous solutions were identified and characterized by the values of the isotropic spectroscopic splitting factor $g_{iso}$ and isotropic hyperfine coupling constant $|A_{iso}|$ measured by means of EPR spectroscopy. In strongly acidic aqueous solutions the only vanadocene species $[Cp_2V(H_2O)_2]^{2+}$ (**III**; $g_{iso}$= 1.9828, $|A_{iso}|$ = 7.92 mT) is present (stable for several days). In strongly acidic 0.103M NaCl solution the prevailing species **III** coexists in equilibrium with $[Cp_2V(H_2O)Cl]^+$ (**II**; $g_{iso}$= 1.9848, $|A_{iso}|$ = 7.68 mT). The latter species **II** was unambiguously identified in concentrated HCl solutions, where it coexists in equilibria with either **I** or **III** depending on the HCl concentration. Vanadocene dihydroxide, $Cp_2V(OH)_2$ (**IV**; $g_{iso}$= 1.9907, $|A_{iso}|$ = 6.285 mT), was the only vanadocene complex identified at physiological and higher pH values. Its EPR signal has completely disappeared after 72h-exposure of the solution to air.

### *MATERIALS AND METHODS*

### Compounds and solutions

Vanadocene dichloride (**I**) was prepared by the published method [17] and purified by anaerobic Soxhlet extraction with chloroform. The purity of **I** was checked by elemental analysis as well as by IR, EPR and UV/VIS spectroscopies. The purified compound was handled under oxygen-free Ar using standard Schlenk techniques and was stored under Ar in sealed vials. $Me_2SO$ (Fluka) was thoroughly dried.

As solvents the standard physiological solutions [Ringer : 6.50 g (0.111 mol) NaCl, 0.14 g (1.88 mmol) KCl, 0.12 g (1.08 mmol) $CaCl_2$, 0.20 g (2.38 mmol) $NaHCO_3$ / 1000ml solution; Ringer-Lock 9.00 g (0.154 mol) NaCl, 0.42 g (5.63 mmol) KCl, 0.24 g (2.16 mmol) $CaCl_2$, 0.20 g (2.38 mmol) $NaHCO_3$, 1.00 g (5.55 mmol) D-glucose / 1000ml solution; Ringer-Tyrod : 8.00 g (0.137 mol) NaCl, 0.20 g (2.68 mmol) KCl, 0.20 g (1.80 mmol) $CaCl_2$, 0.10 g (1.19 mmol) $NaHCO_3$, 0.10 g (1.05 mmol) $MgCl_2$, 0.05 g (0.417 mmol) $NaH_2PO_4$, 1.00 g (5.55 mmol) D-glucose / 1000ml solution; Krebs-Ringer : 6.895 g (0.118 mol) NaCl, 3.355 g (0.048 mol) KCl, 0.277 g (2.50 mmol) $CaCl_2$, 0.163 g (1.18 mmol) $KH_2PO_4$, 0.144 g (1.20 mmol) $MgSO_4$, 2.100 g (0.025 mol) $NaHCO_3$ / 1000ml solution; saline : 6.02 g (0.103 mol) NaCl / 1000ml solution; D-glucose : 41.46 g (0.230 mol) / 1000ml solution], $Me_2SO$ and the therapeutic solvent system [saline (0.103M NaCl) and $Me_2SO$ (9:1 v/v)] were employed. All chemicals were analytical grade. Deionized double-distilled water was used throughout these experiments.

The blood plasma stabilized with sodium hydrogen citrate was purchased from Imuna (Šarišské Michalany, Slovac Republic) and used as received. The whole human blood used for this study was taken from a healthy man (age of 26 years) and stabilized using EDTA.

### Measurements

EPR spectra were recorded on a X-band spectrometer ESR 221 (ZWG Berlin, Germany) at room temperature in time intervals ranging from 150 s up to several hours or even days after introducing **I** into solvent system. The experimental $g_{iso}$ and $A_{iso}$ values determined from recorded EPR spectra by the standard procedure were corrected for the second - order effects [18]. In order to estimate the abundance ratio (in %) of various vanadium(IV) - containing species in a studied solution or solvent system the recorded EPR spectral first derivate curves consisting of two or more superposed eight-line signals were computer-deconvulated into separate ones corresponding to the respective species. Their abudance ratio was then estimated from the ratio of integral signal areas obtained by double integrating the individual deconvoluted first derivative signals.

The pH measurements were performed with a pH meter MV-870 Digital pH-Messgerät using a glass electrode Radelkis OP-0808P. The pH meter was calibrated with buffers Radelkis „K-21“ pH (25°C) = 2.15 and „K-91“ pH (25°C) = 9.21.

For preparing and handling solutions of **I** the special all-glass cell developed in our laboratory was employed which made possible all operations preceding the EPR measurement (such as introduction and dissolution of **I**, adjustment and control of pH concentration, saturation and purging with Ar, sampling and filling EPR capillary tubes) to be made under argon atmosphere. If necessary, the pH adjustments were made with either concentrated NaOH or $HClO_4$.

### RESULTS

In order to identify species arising from **I** in physiological and therapeutic media as well as in blood plasma and human blood it has been taken advantage of the fact - analogously to our previous study [16] - that the complexes of $Cp_2VX_2$, $[Cp_2VLX]^+$, and $[Cp_2VL_2]^{2+}$, where X represents a monoanionic ligand, and L an uncharged unidentate ligand, generate an eight-line EPR spectrum due to the hyperfine coupling of unpaired *d* electron with the $^{51}V$ (I = 7/2; 99.8%) nucleus. These eight-line spectra can be explained using the spin Hamiltonian $\boldsymbol{H} = \beta g_{iso}H\boldsymbol{S} + A_{iso}\boldsymbol{IS}$ for systems with S = 1/2 and applying second order correction.

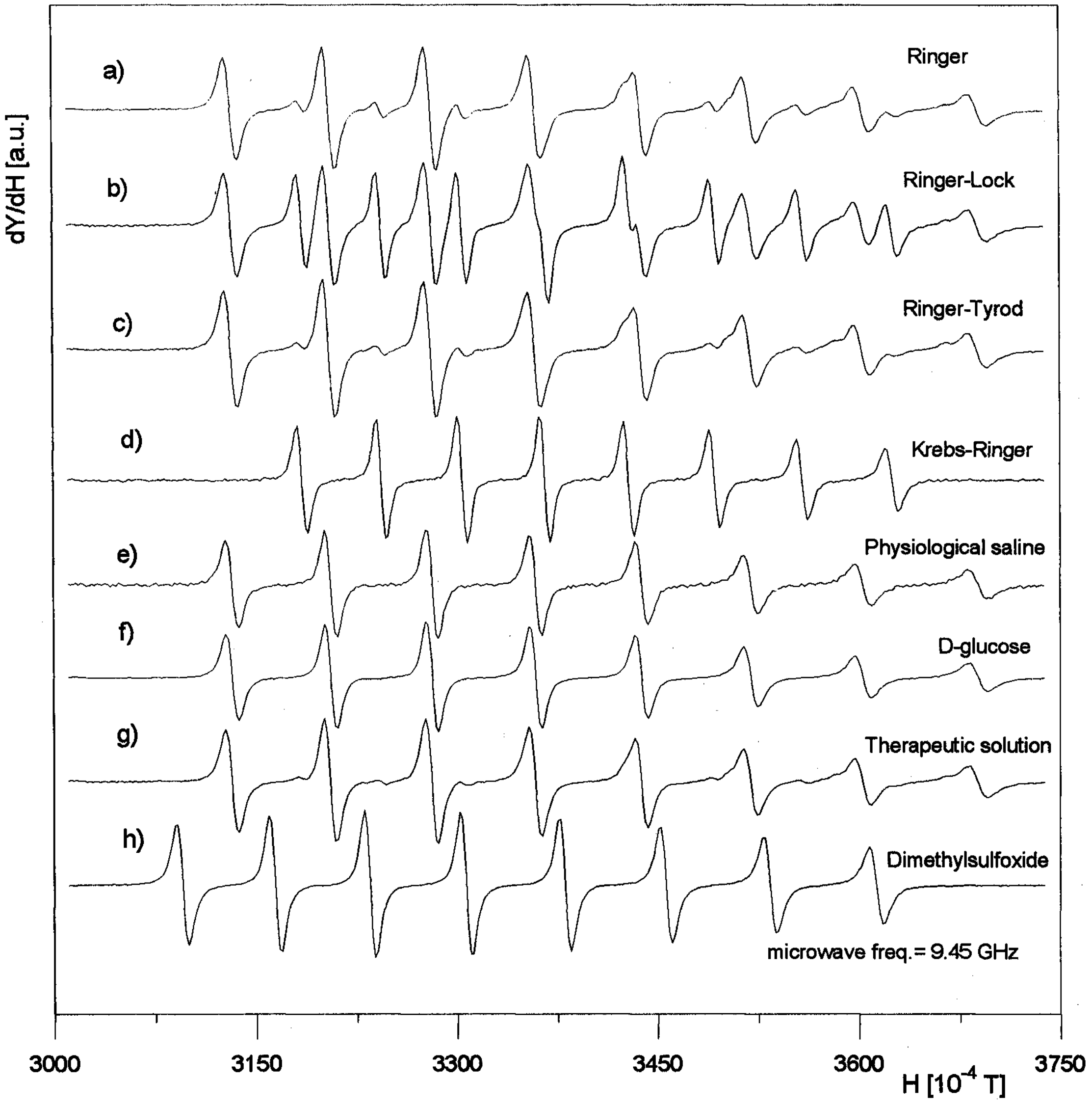

Fig. 1. EPR spectra of vanadocene dichloride in various solutions and solvents (150s after substance introduction into solution; concentration in **I** : $10^{-3}$ M ).

### EPR spectra of I in physiological solutions and in the therapeutic solution DMSO/saline

EPR spectra were measured in both deoxygenated solutions (solvents used were bubbled with pure argon for 30 min at 60°C and the samples for measuring were prepared under argon atmosphere) and in non-deoxygenated solutions (solvents used were not deoxygenated and the samples were prepared under atmospheric air using standard techniques). The solutions for biological and pharmacological experiments were probably used in the latter manner. In the following the deoxygenated solutions treated in the corresponding way will be always meant unless stated otherwise.

### *Ringer solution*

I dissolved in the Ringer physiological solution affords 150s after substance introduction into solution a complex EPR spectrum (Fig.1a) consisting of three eight-line superposed signals belonging to three different vanadocene species. The most intense eight-line signal is characterized by EPR parameters $|A_{iso}|$ = 7.92 mT and $g_{iso}$= 1.981 , both corresponding to the species III (see [16]). The second, less intense eight-line signal with EPR parameters $|A_{iso}|$ = 6.30 mT and $g_{iso}$= 1.991 corresponds to the vanadocene dihydroxide IV. On the last two high-field lines of the signal the presence of an additional weak line could be resolved. The EPR parameters of this third partially resolved eight-line signal, $|A_{iso}|$ = 7.63 mT and $g_{iso}$= 1.985 , can be assigned to the species II as shown previously [16]. 24h after introduction of I into the Ringer solution the traces of the fourth eight-line signal ($|A_{iso}|$ = 11.60 mT, $g_{iso}$= 1.958) appeared. This signal is unambiguously characteristic of vanadyl species VII. The intensity of this signal gets stronger with increasing time.

### *Ringer-Lock solution*

I dissolved in the Ringer-Lock physiological solution displays 150s after substance introduction into solution a complex EPR spectrum consisting - analogously to the preceding case - of three eight-line superposed signals belonging to the three species present (Fig.1b): III ($|A_{iso}|$ = 7.92 mT, $g_{iso}$= 1.983), IV ($|A_{iso}|$ = 6.30 mT, $g_{iso}$= 1.991) and II ($|A_{iso}|$ = 7.63 mT, $g_{iso}$= 1.985). The latter species can be identified through an additional weak line, which is discernible on the last two high-field lines of the spectrum. Also in this case the traces of the fourth eight-line signal ($|A_{iso}|$ = 11.60 mT, $g_{iso}$= 1.958) appeared 24h after introduction of I into the Ringer-Lock solution. This additional signal whose intensity increases with time can be again assigned to the species VII.

### *Ringer-Tyrod solution*

I dissolved in the Ringer-Tyrod solution displays similarly a complex EPR spectrum consisting of three eight-line superposed signals, only differing in the relative abundance of three vanadocene species (Fig.1c). The most abundant species shows to be III ($|A_{iso}|$ = 7.92 mT, $g_{iso}$= 1.983), while the species IV is present in much lesser amount ($|A_{iso}|$ = 6.30 mT, $g_{iso}$= 1.991). A slight amount of the species II ($|A_{iso}|$ = 7.63 mT, $g_{iso}$= 1.985) is also present. Similarly to the preceding cases the traces of the vanadyl species VII appeared in the EPR spectrum 24h after introduction of I into solution ($|A_{iso}|$ = 11.60 mT, $g_{iso}$= 1.958). The signal intensity of the latter species increased with time.

### *Krebs-Ringer solution*

I dissolved in this physiological solution shows - also 150s after introduction of I into solution - the simple eight-line EPR spectrum with magnetic parameters ($|A_{iso}|$ = 6.29 mT, $g_{iso}$= 1.991) corresponding to the species IV (Fig.1d). The measured spetrum remains unchanged in its shape, parameters and intensity for at least 24h.

### *Physiological saline*

I in physiological saline (103 mM NaCl) similarly affords a marked eight-line EPR spectrum corresponding to the diaquavanadocene dication III ($|A_{iso}|$ = 7.92 mT, $g_{iso}$= 1.983) (Fig.1e). The additional line ($|A_{iso}|$ = 7.63 mT, $g_{iso}$= 1.985) superposed on each of the two high-field lines of the eight-line signal gives evidence for the simultaneous presence of a tiny amount of a species **II**. 24h after substance introduction into saline the traces of the third eight-line signal corresponding to the species **VII** ($|A_{iso}|$ = 11.60 mT, $g_{iso}$= 1.958) appeared and increased.

*D-glucose*

**I** in isotonic aqueous D-glucose solution shows the eight-line EPR spectrum corresponding to the species **III** ($|A_{iso}|$ = 7.92 mT, $g_{iso}$= 1.983) (Fig.1f). 24h after introduction of **I** into solution the traces of another eight-line signal of the species **VII** ($|A_{iso}|$ = 11.60 mT, $g_{iso}$= 1.958) appeared. This signal increased in intensity with time.

In order to elucidate the presence of species arising from **I** in all the physiological solutions mentioned above the pH values of solutions before and after introduction of **I** were measured. The pH values are given in Table I.

TABLE I. The pH values of various physiological solutions without and with I

| Physiological solution | without I | with I[a] |
|---|---|---|
| Ringer | 8.1 | 3.4 |
| Ringer-Lock | 7.8 | 3.4 |
| Ringer-Tyrod | 6.4 | 3.3 |
| Krebs-Ringer | 8.9 | 7.1 |
| 0.103 M NaCl | 6.3 | 3.4 |
| D-glucose | 6.4 | 3.2 |

[a] Solution used for EPR measurement

In addition to the EPR spectra measured in deoxygenated physiological solutions the measurements given above have also been carried out in the presence of air oxygen. **I** dissolved in physiological solutions (Ringer, Ringer-Lock, Ringer-Tyrod, Krebs-Ringer, saline and D-glucose) used without prior deoxygenation exhibits the EPR spectra with magnetic parameters corresponding to the species **II**, **III**, **VI** and **VII**.

*DMSO/saline (v/v=1/9) therapeutic solution*

The EPR spectrum of **I** was further investigated in the therapeutic solution (DMSO-saline v/v=1/9) serving for metallocene cytostatic application in preclinical studies. These EPR measurements were carried out 150s, 1h, 24h, 48h and 6d after introduction of **I** into the therapeutic solution in order to ascertain what kinds of vanadocene(IV) or vanadium(IV) species arise, persist and/or disappear. The EPR spectrum recorded 150s after introduction of **I** into the therapeutic solution consists (Fig.1g) of three eight-line signals corresponding to three different species. The prevailing species **III** is characterized by an eight-line signal having magnetic parameters $|A_{iso}|$ = 7.96 mT and $g_{iso}$= 1.983. The second eight-line signal can be assigned to a little amount of **IV** ($|A_{iso}|$ = 6.31 mT, $g_{iso}$= 1.991). The third species, i.e. **II**, becomes evident through weak lines superposed on the last two high-field lines ($|A_{iso}|$ = 7.63 mT, $g_{iso}$= 1.984). The EPR spectrum measured after 1h is identical with the preceding one. After 24h the EPR spectrum shows the presence of the fourth eight-line signal ($|A_{iso}|$ = 11.60 mT, $g_{iso}$= 1.958) of the additional species **VII**. The spectrum of the same solution sample after 48h reveals that the present species are still the same but the signal intensity of **III** decreases in favour of other three species. The spectrum recorded after 6d shows that the signal intensities of four species present in solution are in the following order: **III**>**VII**>**IV**>**II**. The magnetic parameters $|A_{iso}|$ and $g_{iso}$ of respective vanadocene species are time independent.

150s after dissolving **I** in the non-deoxygenated therapeutic solvent, the EPR spectrum exhibits four simple eight-line signals corresponding to four various vanadium species. The prevailing species is **III**

($|A_{iso}|$ = 7.96 mT, $g_{iso}$= 1.983). The other species **VII** ($|A_{iso}|$ = 11.60 mT, $g_{iso}$= 1.958), **IV** ($|A_{iso}|$ = 6.31 mT, $g_{iso}$= 1.991) and **II** ($|A_{iso}|$ = 7.63 mT, $g_{iso}$= 1.984) are present in minor amounts, the latter being only discernible through weak lines superposed on the last two intense high-field lines. This system was also investigated at five different time intervals (150s, 1h, 24h, 48h, 6d) after sample preparation. The spectrum recorded after 1h consists of three eight-line signals. On the basis of their magnetic parameters these three signals are assigned to the following species: **IV** with unchanged signal intensity, **VII** with increased signal intensity and **III** having the most intense signal. With increasing time the signal intensity of **VII** increases to the detriment of **III**. The signal intensity of **IV** remains unchanged. 48h after mixing **I** with the therapeutic solvent the signal intensities of the species **VII** and **III** become equalized. **IV** is only present in a minimum amount. The $|A_{iso}|$ and $g_{iso}$ parameters are time independent. 6 d after introduction of **I** into the non-deoxygenated therapeutic solvent the only vanadium(IV) species present in examined solution is **VII** as evidenced by the single eight-line signal exhibiting characteristic magnetic parameters $|A_{iso}|$ = 11.60 mT and $g_{iso}$= 1.958.

***DMSO***

The EPR spectrum of **I** was also measured in neat DMSO which serves as a minor, auxiliary component of the therapeutic solvent system used for preclinical animal tests as mentioned above. The time dependence of the formation of various vanadocene and vanadium species was again monitored in thoroughly deoxygenated DMSO. 150s after introduction of **I** into DMSO the simple eight-line EPR spectrum was found (Fig.1h). The observed magnetic parameters ($|A_{iso}|$ = 7.43 mT, $g_{iso}$= 1.988) correspond to those of **I** itself (for comparison: **I** in $CHCl_3$ exhibits $|A_{iso}|$ = 7.43 mT, $g_{iso}$= 1.9882 [17]). The EPR spectrum of the same sample after 5min seems at first sight to be a simple eight-line signal having the same magnetic parameters as that recorded after 150s. However, a careful examination of the spectrum allows to detect additional lines superposed on the last two high-field lines and belonging apparently to the additional eight-line signal of low intensity. The intensity of this signal having $|A_{iso}|$ = 7.68 mT and $g_{iso}$= 1.98 increases with time. The $|A_{iso}|$ and $g_{iso}$ values of this new signal are conspicuously different from those of **I** as well as from those of other vanadocene species hitherto described. We assign this newly appeared EPR signal tentatively to the complex cation $[Cp_2VCl(DMSO)]^+$ (its $|A_{iso}|$ and $g_{iso}$ values are very close to those of **II**). 24h after introduction of **I** into DMSO an EPR spectrum is obtained consisting of two overlapping eight-line signals. The present species are still the same except for the signal of **I** decreasing in intensity in favour of $[Cp_2VCl(DMSO)]^+$. The spectrum of **I** in DMSO, measured after 3d, is identical with the preceding (24h) one.

The above time dependence was also investigated in the presence of air oxygen. The obtained isotropic EPR spectra consist of two eight-line signals corresponding to two different species. Undoubtedly, the first species was **I** ($|A_{iso}|$ = 7.44 mT, $g_{iso}$= 1.988). Another species ($|A_{iso}|$ = 11.78 mT, $g_{iso}$= 1.964) appeared immediately after introducing **I** into DMSO and its signal increased with increasing time. The EPR spectrum recorded after 24h was a simple eight-line signal corresponding only to one species. Its magnetic parameters are different from those of **I** as well as of all other species hitherto identified. The higher $|A_{iso}|$ value indicates the presence of a vanadyl species. We assign this eight-line EPR signal tentatively to the cation $[VO(DMSO)_n]^{2+}$ (for comparison, we have measured the EPR spectrum of $VOSO_4$ in DMSO: its eight-line signal exhibits $|A_{iso}|$ = 11.05 mT and $g_{iso}$= 1.969).

### EPR spectra of I in blood plasma and whole blood

Biological experiments (see above) pointed to the nucleic acids, especially DNA of cellular nucleus, as probable primary molecular target for antitumor active metallocene dihalides including **I**. Before the metallocene molecule reaches the tumor cell, it has to pass the blood bed after its application in therapeutic solution. In order to determine which vanadocene species does (or do) exist after introducing **I** - (DMSO/0.103M NaCl) into the blood bed we will now try to clarify the nature of interaction **I** - blood plasma and **I** - whole human blood.

The blood plasma and the therapeutic solution were in no way treated (for example deoxygenated). The preparation of samples for EPR measurements was carried out using standard techniques without inert atmosphere.

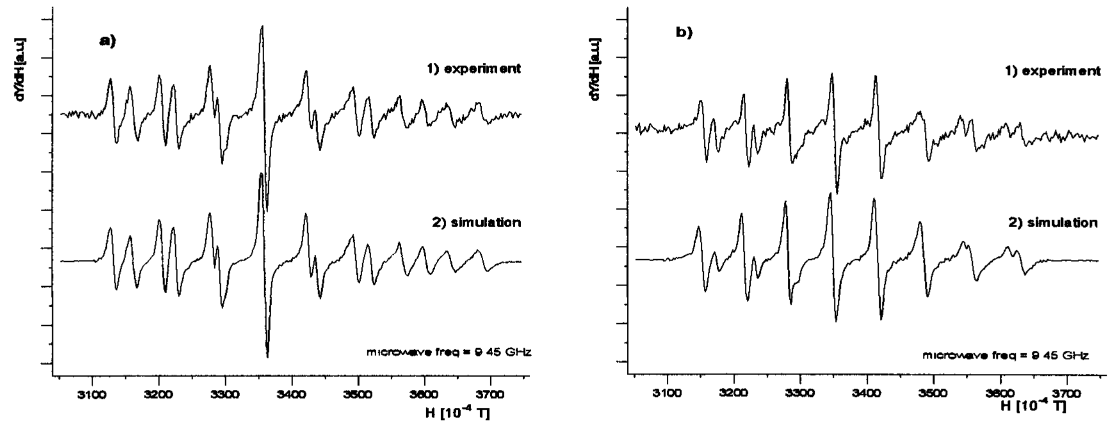

Fig. 2. The EPR spectrum of the system I - blood plasma at a) pH = 3.71, b) pH = 7.13 ( concentration in I: $10^{-4}$ M ).

***Blood plasma - I***

The series of solutions **I** - blood plasma were investigated in the pH range 0.12 - 9.13. The region of physiological pH values was examined most thoroughly. The studied solutions give (250s after introduction of **I** into solution and adjustment of pH value using 1M $HClO_4$ or 1M NaOH) complex EPR spectra consisting of several simple eight-line signals of vanadocene species.

**I** in blood plasma solutions having final pH values 0.12 - 3.35 shows a composite EPR spectrum consisting of two eight-line signals (Fig.2a). The signal of markedly higher intensity with magnetic parameters $|A_{iso}|$ = 7.92 mT, $g_{iso}$= 1.983 can be unambiguously assigned to the cation **III**. The other eight-line signal whose intensity increases with increasing pH is characterized by magnetic parameters $|A_{iso}|$ = 6.87 mT, $g_{iso}$= 1.984, which differ from those of all the vanadocene species studied till now. We assign this new signal to a complex "vanadocene-citrate". This complex species arises probably through reaction of **I** (or of another vanadocene species) with sodium hydrogen citrate used to stabilize the blood plasma. For comparison we measured EPR spectrum of **I** in aqueous sodium hydrogen citrate. This solution (pH=4.2) displays 250s after introduction of **I** an EPR spectrum consisting of two eight-line signals corresponding to two species. The less intensive signal having $|A_{iso}|$ = 7.92 mT, $g_{iso}$= 1.983 corresponds to the cation **III**, the other more intensive signal has magnetic parameters ($|A_{iso}|$ = 6.87 mT, $g_{iso}$= 1.984) identical with those of the species found in the system **I** - blood plasma and designated as "vanadocene-citrate".

The systems **I** - blood plasma with final pH values in the range from 3.35 to 7.13 exhibit complex EPR spectra consisting of three eight-line signals. The first signal with intensity decreasing with increasing pH and magnetic parameters $|A_{iso}|$ = 7.92 mT, $g_{iso}$= 1.983 can be assigned to the cation **III**. The second eight-line signal, whose intensity slightly decreases at limiting pH values of the given interval, corresponds to the "vanadocene-citrate" species ($|A_{iso}|$ = 6.87 mT, $g_{iso}$= 1.984). The third eight-line signal with intensity increasing with increasing pH has magnetic parameters $|A_{iso}|$ = 6.29 mT, $g_{iso}$= 1.991, corresponding therefore to **IV**.

The EPR spectra of the system **I** - blood plasma with pH values in the range from 7.13 to 9.13 exhibit two superposed eight-line signals (Fig.2b). The first signal with intensity decreasing rapidly with increasing pH belongs to the "vanadocene-citrate" species ($|A_{iso}|$ = 6.87 mT, $g_{iso}$= 1.984), the other signal ($|A_{iso}|$ = 6.29 mT, $g_{iso}$= 1.991), the intensity of which raises with increasing pH, can be unequivocally assigned to the **IV**.

***Blood plasma - I/therapeutic solution***

**I** dissolved in the therapeutic solution (DMSO +103 mM NaCl, 1:9 v/v) and added to the blood plasma affords a solution of final pH= 6.32 , which 150s after mixing exhibits an EPR spectrum consisting of two simple eight-line signals. The more intense signal having $|A_{iso}|$ = 6.87 mT, $g_{iso}$= 1.984 corresponds to the "vanadocene-citrate" species, the less intense one to the species **IV** ($|A_{iso}|$ = 6.29 mT, $g_{iso}$= 1.992).

***Whole blood - I***

150s after introduction of **I** into human blood stabilized using disodium EDTA one obtains the simple eight-line EPR spectrum corresponding to the single species **IV**.

***Whole blood - I/therapeutic solution***

**I** dissolved in the therapeutic solution and added to the whole blood stabilized using disodium EDTA exhibits (after 250s) the simple eight-line EPR spectrum whose magnetic parameters $|A_{iso}|$ = 6.285 mT, $g_{iso}$= 1.991 corresponds unequivocally to the species **IV**.

### DISCUSSION

The prerequisite for elucidating the nature of interactions **I** - biomolecule in extracellular medium is the detailed knowledge of the behaviour of **I** in various physiological solutions as well as in the therapeutic solution used for *in vivo* applications. Considering that the physiological solutions are essentially the aqueous ones, it is possible to take advantage of the scheme I for the behaviour of **I** in water in order to discuss the vanadocene species present [15,16].

On the basis of application modes of **I** in physiological or therapeutic solutions mentioned in the literature it may be presumed that the applied solutions were not deoxygenated. Therefore it was necessary to investigate the behaviour of **I** both in deoxygenated and non-deoxygenated solutions.

As it follows from our studies of **I** in physiological solutions, the existence of species **II**, **III**, **IV**, and **VII** have been proven. These species exhibit the magnetic parameters identical with those of the species recorded in aqueous solutions of **I** at various pH values and at various chloride ion concentrations [16]: **II** (high $Cl^-$ concentration), **III** (pH = 0-7), **IV** (pH = 3.1-11.9), and **VII**. All of the species exhibit eight-line EPR signals characteristic of the hyperfine *d*-electron - $^{51}V$ nucleus coupling. The magnetic parameters of vanadocene(IV) or vanadium(IV) species identified by EPR are summarized in Table II.

TABLE II. The magnetic parameters of vanadocene or vanadium species

| | $|A_{iso}|$ [mT] | $g_{iso}$ |
|---|---|---|
| $Cp_2VCl_2$(I) | 7.43 | 1.988 |
| $[Cp_2V(H_2O)Cl]^+$ (II) | 7.68 | 1.985 |
| $[Cp_2V(H_2O)_2]^{2+}$(III) | 7.92 | 1.983 |
| $Cp_2V(OH)_2$(IV) | 6.285 | 1.991 |
| $[Cp_2VCl(DMSO)]^+$ (V) | 7.69 | 1.985 |
| $[VO(DMSO)_5]^{2+}$(VI) | 10.78 | 1.964 |
| $[VO(H_2O)_5]^{2+}$(VII) | 11.56 | 1.955 |
| "vanadocene-citrate" | 6.87 | 1.984 |

The composite EPR spectra consisting of several superposed eight-line signals were resolved into separate eight-line components. The abundance ratio of individual vanadocene and vanadium(IV) species formed by dissolving **I** in a given solution was estimated as described in Materials and Methods. The resulting data are summarized in Table III.

TABLE III. The abundance ratio of individual vanadocene and vanadyl species formed by dissolving **I** in a physiological solution

| I + phys. solution t =150s[a] (24h)[a] | I [%] | II [%] | III [%] | IV [%] | VII [%] |
|---|---|---|---|---|---|
| Ringer | 0 ( 0 ) | 11 ( 8 ) | 79 ( 72 ) | 10 ( 12 ) | 0 ( 8 ) |
| Ringer-Lock | 0 ( 0 ) | 7 ( 4 ) | 49 ( 47 ) | 44 ( 45 ) | 0 ( 4 ) |
| Ringer-Tyrod | 0 ( 0 ) | 18 ( 14 ) | 75 ( 74 ) | 7 ( 7 ) | 0 ( 4 ) |
| Krebs-Ringer | 0 ( 0 ) | 0 ( 0 ) | 0 ( 0 ) | 100 ( 100 ) | 0 ( 0 ) |
| Physiological saline | 0 ( 0 ) | 17 ( 16 ) | 83 ( 82 ) | 0 ( 0 ) | 0 ( 2 ) |
| D-glucose | 0 ( 0 ) | 0 ( 0 ) | 100 ( 96 ) | 0 ( 0 ) | 0 ( 4 ) |

[a] t = time of measurement after introduction of **I**

In the deoxygenated Ringer solution (Fig.1a) being used for studies with cold-blooded animals the species **III**, **IV**, and **II** (only traces) with magnetic parameters given in Table II were identified. In the deoxygenated Ringer-Lock and Ringer-Tyrod solutions the same species were present although in slightly differing relative amounts. These experimental findings can be explained on the basis of the virtually identical pH values attained after dissolving **I**. The species **IV** alone is present in the deoxygenated Krebs-Ringer solution (Fig.1d) which agrees with the previous measurement [16] showing the presence of the only species **IV** in solutions of pH values 7.1 - 11.88. This is the only physiological solution studied which attains a higher pH value, i.e. pH = 7.1 , after dissolving **I**. The deoxygenated saline - **I** solution contains the prevailing species **III** in addition to a small amount of **II**. The last of all examined deoxygenated solutions, the isotonic solution D-glucose-**I** exhibits the presence of a single species, **III** (Fig.1f ). All of the deoxygenated physiological solutions of **I** afford EPR spectra which are constant in form, magnetic parameters and intensities for at least 24 hours. With the exception of the Krebs-Ringer solution all of the other solutions of **I** reveal a trace amount of **VII** after 24 h.

**I** dissolved in non-deoxygenated physiological solutions affords EPR spectra after 150s exhibiting the presence of the species **II**, **III**, **IV**, and **VII**. In contrast to the EPR spectra of **I** in deoxygenated solutions (150s after introducing **I** into solution), the eight-line signal characteristic of the pentaaquavanadyl cation **VII** is observed in this case. The abundance ratios of all species correspond - with minute deviations - to those of these species formed in the corresponding deoxygenated physiological solution 24h after mixing (Table III).

Next the **I** - therapeutic solution system was investigated. This therapeutic solution used for preclinical testing consists of DMSO and saline (1:9 v/v). DMSO acts as auxiliary solvent increasing the solubility of **I**. In view of the fact that the therapeutic solution consists of two different components, it is necessary to investigate the interaction of **I** with either components separately. Since the hydrolysis behaviour of **I** in saline is already known, it was also necessary to investigate the interaction of **I** in the other, though minor component of the therapeutic solution, i.e. DMSO.

By means of EPR, only two species have been found in the deoxygenated water-free DMSO mixed with **I**. **I** affords simple eight-line EPR signal of the intact **I** measured 150s after mixing **I** with DMSO. Already after 5 min yet one additional eight-line signal appears in addition to the signal of **I**. The intensity of this additional signal increases with time and its magnetic parameters differ significantly from those of all other vanadocene species hitherto identified. On the basis of the arguments given below this signal has been assigned to the $[Cp_2VCl(DMSO)]^+$ species (**V**). The EPR spectrum of **I** in non-deoxygenated DMSO consists of two signals, one corresponding to **I** and the other probably to the $[VO(DMSO)_n]^{2+}$ species. This new signal appeared immediately after mixing **I** with DMSO (150s) and its intensity raised with time. After 24h the $[VO(DMSO)_n]^{2+}$ species was the only one present.

From EPR measurements in the deoxygenated and non-deoxygenated DMSO we can draw the following conclusions: (i) In the absence of air oxygen the substitution of one $Cl^-$ ligand for the DMSO molecule occurs in the course of time

$$Cp_2VCl_2 + DMSO \rightleftharpoons [Cp_2VCl(DMSO)]^+ + Cl^- .$$

The coordination of one DMSO molecule resembles that of one $H_2O$ molecule during dissolving **I** in a water solution of a high $Cl^-$ concentration:

$$Cp_2VCl_2 + H_2O \rightleftharpoons [Cp_2V(H_2O)Cl]^+ + Cl^- .$$

The close similarity can be observed between the two vanadocene monochloride cationic species, which is due to the fact that one O-donor is always coordinated to the vanadium center through the O-atom in both species. (ii) In the presence of air oxygen the two $Cp^-$ ligands of **I** are displaced to give perhaps a vanadyl-DMSO complex, having probably the $[VO(DMSO)_5]^{2+}$ (**VI**) formula corresponding to the analogous aqua complex $[VO(H_2O)_5]^{2+}$ (**VII**). In view of the fact that these two vanadyl species contain six oxygen donor atoms in the first coordination sphere of the V center, the similarity of their $g_{iso}$ and $A_{iso}$ values (Table II) is not surprising. The identity of the $[VO(DMSO)_5]^{2+}$ (**VI**) species is supported by the ESR spectrum of the vanadyl sulphate-DMSO solution which displays one eight-line signal with magnetic parameters identical with those of the $[VO(DMSO)_5]^{2+}$ species presumed to be present in the **I**-DMSO under exposure to air.

Three species, i.e. **II**, **III**, and **IV**, have been observed in the deoxygenated therapeutic solution saline/DMSO (Fig.1g) 150s after introduction of **I**. Neither $[VO(DMSO)_5]^{2+}$ (**VI**) nor $[Cp_2VCl(DMSO)]^+$ (**V**) have been observed. 24h after introducing **I** into solution the signal of the $[VO(H_2O)_5]^{2+}$ species appeared in the EPR spectrum. Its intensity raises with increasing time whereas the intensities of other signals decrease. **I** in the therapeutic solution under conditions used for both *in vivo* and *in vitro* biological experiments exhibits EPR spectra consisting of eight-line signals corresponding to the species **II**, **III**, **IV**, and **VI**. These four species are already present 150s after introducing **I** into the therapeutic solvent system. The signal corresponding to the cation **II** disappeared within 1h. Also the intensity of the complex **IV** diminishes so that it is only present in trace amounts after 48h. On the other hand, the intensity of the signal corresponding to the cation **VII** raises with increasing time while that of the cation **III** simultaneously drops. Apparently the analogous mechanism applies here as for the reaction **I** - non-deoxygenated water (pH = 0-3.0). The sole species **VII** is observable 6 days after introducing **I** into non-deoxygenated therapeutic solvent system. The species **V** and **VI** found both in non-deoxygenated and deoxygenated DMSO were not observed in the non-deoxygenated therapeutic solvent system. The dominant component (i.e. $H_2O$ + NaCl) of the used therapeutic solvent system is the only component which is coordinatively effective in the interaction with **I** in an experimentally interceptible manner in contrast to the minor component (DMSO).

Now we will try to answer the following question: what specific vanadocene(IV) or vanadium(IV) species will be present in the blood plasma and the whole blood after application of antitumor active **I** ? Since both the blood plasma and the blood represent essentially the aqueous systems, it is possible to use the known hydrolysis scheme of **I** for discussion of the relevant species. In order to ascertain the nature of resulting species we may get out of the behaviour of **I** in physiological solutions. The composition of the used physiological solutions namely mimics the mineral composition of the blood and the plasma, as well. All of the measured EPR spectra were resolved into separate simple eight-line signals. The EPR spectra of the vanadocene species present in the **I**-blood plasma systems having various resulting pH values are shown in Figs. 2a,b.

The EPR measurements of **I** in blood plasma solutions of various resulting pH values ranging from 0.12 to 9.13 have shown the existence of the vanadocene species **III**, **IV**, and "vanadocene-citrate". The species **III** can be found in the pH range 0.12 - 7.13 in excellent agreement with results of EPR

measurements of **I** in aqueous solutions[16], where this species appears in the pH range 0 - 7.1. The complex **IV** can be identified in I-blood plasma systems having pH values 3.39 - 9.13. We find again an excellent agreement with results obtained for aqueous solutions exhibiting the existence of **IV** in the pH range from 3.1 to 11.9. In addition to the EPR signals of the species **III** and **IV** we observed an additional eight-line signal in the **I** - blood plasma system corresponding to a novel vanadocene species, probably a complex "vanadocene-citrate". The nature of this novel vanadocene species may be elucidated by means of the EPR spectrum of **I** in aqueous sodium hydrogen citrate. The eight-line EPR signal of that solution displays magnetic parameters $g_{iso}$ and $A_{iso}$ identical with those of the above "vanadocene-citrate" species. It should be remembered that the sodium hydrogen citrate is used to stabilize the blood plasma.

**I** dissolved in the therapeutic solvent system and added to the blood plasma solution affords the composite EPR spectrum consisting of two eight-line signals corresponding to the species **IV** and "vanadocene-citrate".

For next investigation of interactions of **I** in body fluids we tried to avoid the use of the sodium hydrogen citrate as stabilizer which is not present in the human body in such a high concentration as used for blood plasma stabilization. That was why the whole blood was stabilized using EDTA, which does not react with any relevant vanadocene species. EDTA only reacts with the vanadyl species **VII**. **I** dissolved in the stabilized whole blood shows the single eight-line EPR signal corresponding to the complex **IV**. **I** dissolved in the therapeutic solvent system and subsequently added to the stabilized whole blood exhibits the same EPR spectrum demonstrating the presence of the complex **IV** alone.

### *CONCLUSIONS*

Metallocene dichlorides, $(\eta^5\text{-}C_5H_5)_2MCl_2$ , (M = Ti, V, Nb, Mo), represent a novel class of very active antitumor agents. In order to reveal the nature of their molecular action it is necessary to know which particular species enters the tumor cell and interacts with target organelles, biostructures and biomolecules. Metallocene dichlorides are not stable in aqueous media. They undergo equilibrium aquation, dissociation and hydrolysis reactions. The knowledge of their behaviour in aqueous media is of fundamental importance since they meet with the water both at injection application in physiological media and after entering into biological systems including the tumor cell.

Compound **I** behaves in most of the physiological solutions, i.e. in solutions the composition and actual pH value of which mimic those of the blood serum or other body fluids, analogously to its behaviour in aqueous solutions of corresponding pH value. The hydrolysis products of the **I** do not therefore react with any inorganic or organic component of the used physiological solutions in the first coordination sphere of the central vanadium atom. The stability of the vanadocene ring-V-ring framework in non-deoxygenated, simple aqueous ones (c.f. ref.[16]). This stability difference is most remarkable at physiological pH (~7). when compound **I** is dissolved in a Krebs-Ringer physiological solution (pH=7.2), one EPR signal characteristic of the species **IV** is observed. This signal shows no decrease of intensity indicating a reaction with air oxygen even after 24h.

In the therapeutic solvent system under conditions corresponding to *in vivo* and *in vitro* biological experiments and preclinical tests, but with exclusion of air access, **I** behaves analogously to **I** in saline (0.103M NaCl). This means that DMSO does not produce any other vanadocene species than that expected in saline itself. Therefore, we can conclude the role of DMSO to lie only in improving the solubility of the introduced **I**.

In order to determine which vanadocene species may exist if **I** (or **I** dissolved in the therapeutic solvent system) is introduced into the blood plasma or the whole blood. It was possible to use the knowledge of the behaviour of **I** in aqueous and physiological solutions, since the plasma and blood can be regarded as essentially aqueous solutions despite their system complexity. We can draw one very important conclusion: compound **I** does not react with any blood plasma / blood components in the first coordination sphere of the vanadium centre except for the water. Its behaviour in these biological systems corresponds to the reactions already described in the case of aqueous solutions of the corresponding pH value. This means that the single vanadocene species **IV** is present after applying **I** into whole blood as was anticipated by Toney and Marks[15]. The species **IV** does not react with any blood plasma / blood component through any reaction

(e.g. substitution reaction) which would take place in the first (inner) coordination sphere of the central vanadium atom. The possibility of some reactions of **IV** which would take place in the second (outer) coordination sphere is not excluded. However the identification of such a second (outer) sphere interaction by means of magnetic parameters $g_{iso}$ and $A_{iso}$ is probably impossible, since it is the first (inner) coordination sphere of a given vanadocene or vanadium $d^1$ species which determines its $g_{iso}$ and $A_{iso}$ values. The effect of changes in the second sphere on these values is very small and probably hardly perceptible.

### ACKNOWLEDGEMENTS

We are indebted to the Grant Agency of the Czech Republic (Grants No.203/94/0024 and 203/97/0502) for generous support of this work.

### REFERENCES

1.Köpf-Maier,P.; Köpf,H., *Chem.Rev.* **1987**, 87, 1137.

2.Köpf-Maier,P.; Köpf,H., *Struct.Bonding* **1988**, 70, 103.

3. Köpf-Maier,P.; Köpf,H., *Z.Naturforsch.* **1979**, 34b, 805.

4. Köpf-Maier,P.; Köpf,H., *Cancer Chemother.Pharmacol.* **1981**, 5, 237.

5. Toney,J.H.; Rao,L.N.; Murthy,M.S.; Marks,T.J., *J.Breast Cancer Res.Treat.* **1985**, 6, 185.

6. Murthy,M.S.; Toney,J.H.; Rao,L.N.; Kuo,L.Y.; Marks,T.J., *Proc.Am.Assoc.Cancer Res.* **1986**, 27, 279.

7. Murthy,M.S.; Rao,L.N.; Kuo,L.Y.; Toney,J.H.; Marks,T.J., *Inorg.Chim.Acta* **1988**, 152, 117.

8. Köpf-Maier,P.; Herman,G., *Virchows Arch. [Cell Pathol.]* **1984**, 47, 107.

9. Köpf-Maier,P.; Krahl,D., *Naturwiss.* **1981**, 68, 273.

10. Köpf-Maier,P.; Krahl,D., *Chem.Biol.Interact.* **1983**, 44, 317.

11. Köpf-Maier,P.; Wagner,W.; Köpf,H., *Naturwiss.* **1981**, 68, 272.

12. Köpf-Maier,P.; Wagner,W.; Liss,E., *J.Cancer Res.Clin.Oncol.* **1983**, 106, 44.

13. Köpf-Maier, P.; Wagner,W.; Liss,E., *J.Cancer Res.Clin.Oncol.* **1981**, 102, 21.

14. Köpf-Maier, P., *J.Cancer Res.Clin.Oncol.* **1982**, 103, 145.

15. Toney,J.H.; Marks,T.J., *J.Am.Chem.Soc.* **1985**, 107, 947.

16. Pavlík,I.; Vinklárek,J., *Eur.J.Solid State Inorg.Chem.* **1991**, 28, 815.

17. Wilkinson,G.; Birmingham J.M., *J.Am.Chem.Soc.* **1954**, 76, 4281.

18. Rogers,R.N.; Pake,G.E., *J.Chem.Phys.* **1960**, 33, 1107.

**Received: April 14, 1997 - Accepted: May 15, 1997 - Received in revised camera-ready format: June 6, 1997**

